# Local recurrence of sclerosing mucoepidermoid carcinoma with eosinophilia in the upper lip: a case report

**DOI:** 10.1186/s13256-015-0525-8

**Published:** 2015-02-24

**Authors:** Yoshikazu Kobayashi, Koji Satoh, Takako Aizawa, Makoto Urano, Makoto Kuroda, Hideki Mizutani

**Affiliations:** 1 Department of Oral and Maxillofacial Surgery, Fujita Health University School of Medicine, 1-98 Dengakugakubo, Kutsukake, Toyoake, Aichi Pref Japan; 2 Department of Diagnostic Pathology, Fujita Health University School of Medicine, 1-98 Dengakugakubo, Kutsukake, Toyoake, Aichi Pref Japan

**Keywords:** Lip cancer, Minor salivary glands, Sclerosing mucoepidermoid carcinoma, Tissue eosinophilia

## Abstract

**Introduction:**

Sclerosing mucoepidermoid carcinoma with eosinophilia is a rare morphological variant of thyroid carcinoma associated with Hashimoto’s disease. To date, only three such tumors have been reported in the minor salivary glands. We describe the first case, to the best of our knowledge, of recurrent sclerosing mucoepidermoid carcinoma with eosinophilia in the minor salivary glands of the upper lip.

**Case presentation:**

A 61-year-old Japanese man was referred to our hospital with a mass in his median upper lip of four years’ duration. An examination of his median upper lip revealed a well-defined tumor measuring 9×12mm in diameter, which was subsequently resected. Three years after the first surgery, the tumor recurred and was resected. Both tumors were confirmed by histopathology to be sclerosing mucoepidermoid carcinoma with eosinophilia. Neither recurrence nor metastasis was observed in three and a half years of follow-up after the second surgery.

**Conclusion:**

Our findings indicate that sclerosing mucoepidermoid carcinoma with eosinophilia can originate in the minor salivary glands and may be clinically or pathologically misdiagnosed as other conditions.

## Introduction

In 1991, sclerosing mucoepidermoid carcinoma with eosinophilia (SMECE) was proposed to be a rare morphological variant of thyroid carcinoma associated with Hashimoto’s disease [1]. Approximately 30 cases involving the thyroid have been reported in the literature. Similar to thyroid lesions, SMECE occurring in the salivary glands generally has a lower malignancy and better prognosis than conventional mucoepidermoid carcinoma (MEC). To date, only three of these tumors have been reported in the minor salivary glands. Here, we report the case of a 61-year-old Japanese man with recurrent SMECE in his median upper lip and present a review of the relevant literature.

## Case presentation

A 61-year-old Japanese man was referred to our hospital with a mass in his median upper lip of four years’ duration. He had received interferon-ribavirin combination therapy for chronic hepatitis C several years earlier. He had no history of any other illness.

**Figure 1:**
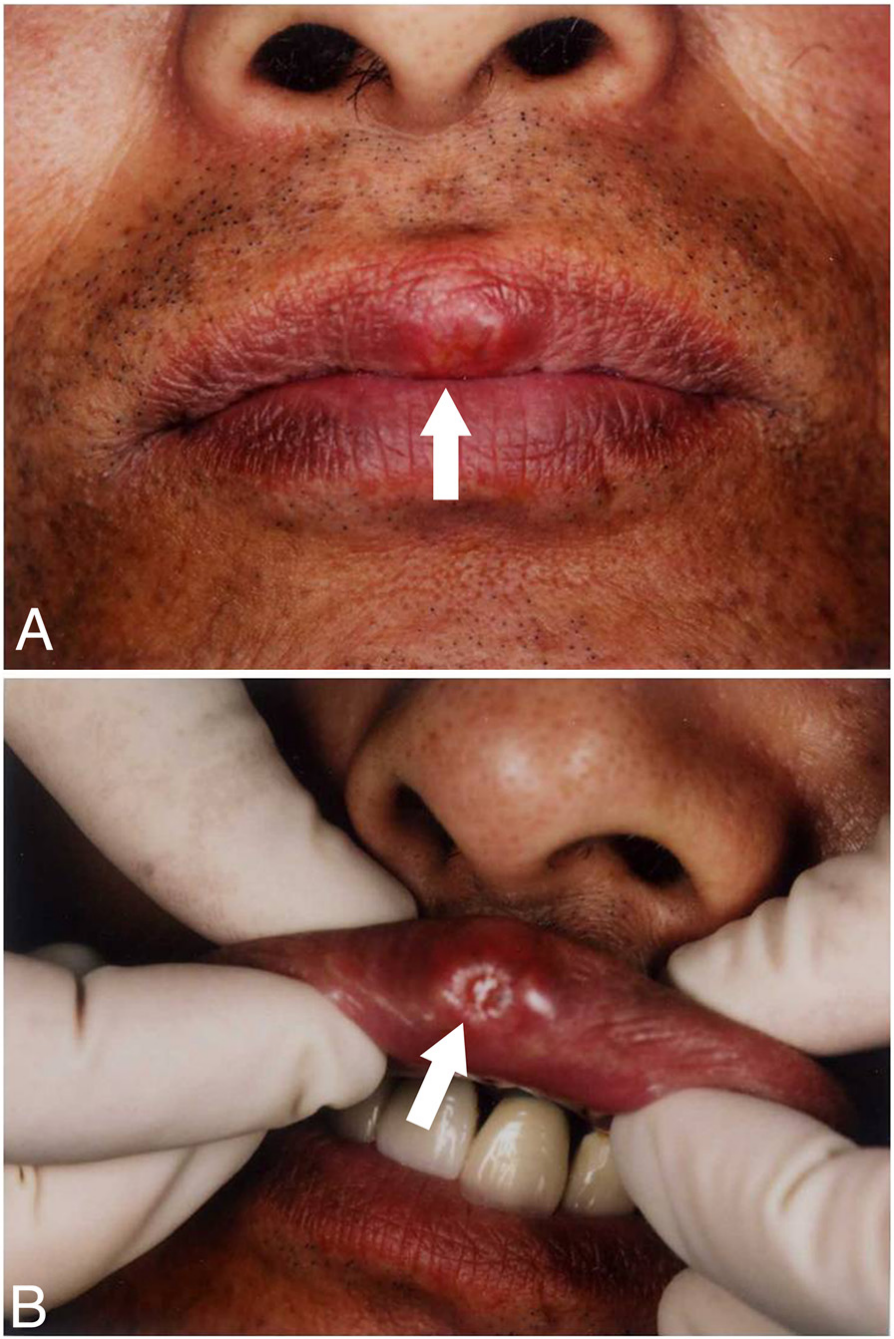
**Gross appearance of the primary tumor. (A)** The tumor was located in the median upper lip, was well-defined, measured 9×12mm in diameter, and was elastic and hard. **(B)** Partial ulceration of the mucous membrane was evident.

Our patient was aware of the mass, which had slowly grown over several years with occasional epithelial detachment or bleeding; however, he never sought treatment. The tumor was located in his median upper lip, was well-defined, measured 9×12mm in diameter, and was elastic, hard and ulcerated (Figure 1). Regional lymph nodes were not palpable.

The tumor appeared to be benign from its appearance and growth rate and was resected under local anesthesia. Preoperative imaging such as computed tomography (CT) or magnetic resonance imaging was not performed because the boundary of the lesion was clear. The tumor surface was smooth and covered with a capsule-like structure, and its cut surface was solid and pale yellow. We assumed that it was a benign tumor such as an atheroma or pleomorphic adenoma.

**Figure 2:**
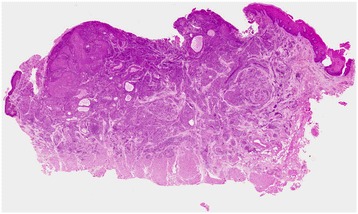
**Macroscopic view of the primary tumor (hematoxylin and eosin staining).** Small neoplastic nests surrounded by markedly hyperplastic fibrous connective tissue were evident. The lining of this specimen consisted of normal muscular tissue.

**Figure 3:**
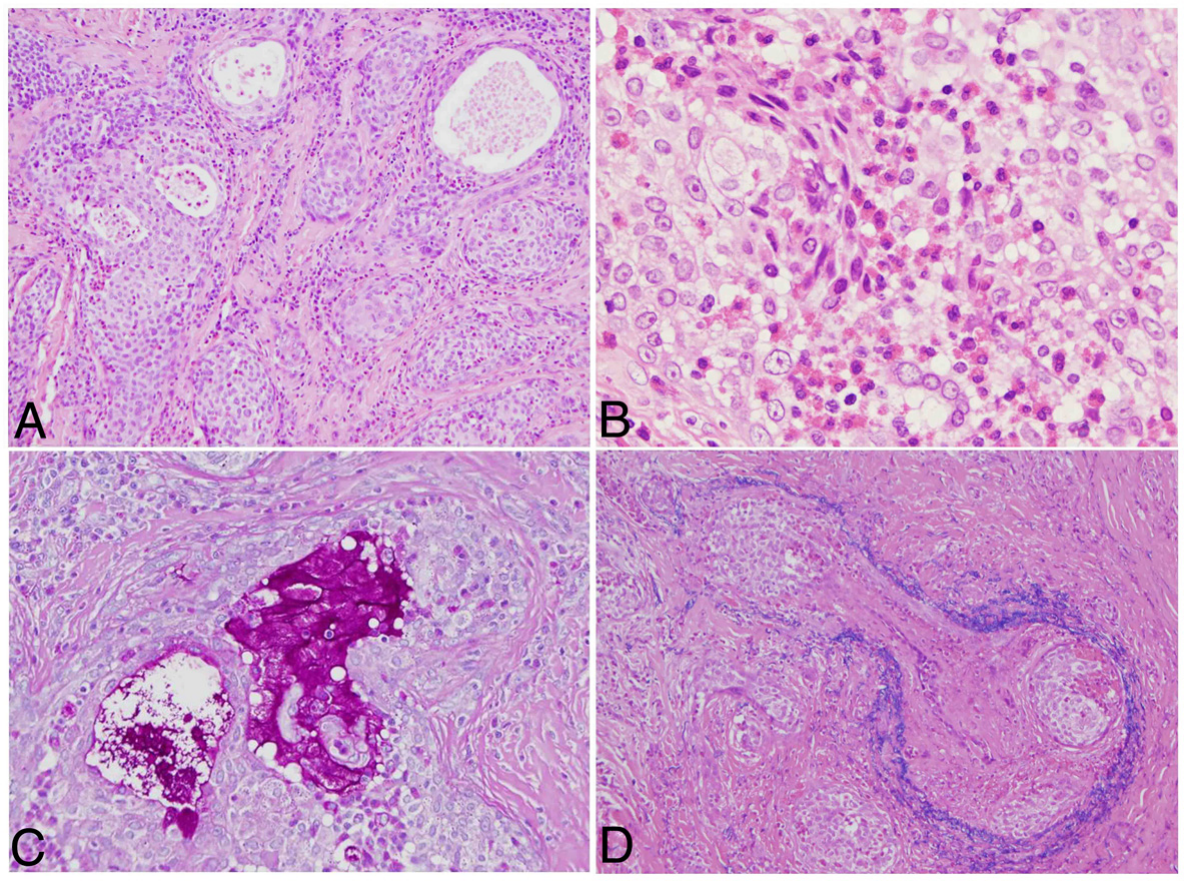
**Histological and immunohistochemical features of the tumor. (A)** Tumor nests consisted of epithelial cells and pseudoglandular structures (hematoxylin and eosin (H&E) staining; ×40). **(B)** Marked eosinophilic infiltration in the tumor stroma (H&E; ×200). **(C)** The tubular structure contained periodic acid–Schiff-positive mucus in the lumen and goblet cells (×100). **(D)** Venous invasion can also be observed (Victoria blue–H&E; ×100).

Beneath the erosive epithelium, small neoplastic nests surrounded by markedly hyperplastic fibrous connective tissue had formed. The tumor cells had pale eosinophilic cytoplasm and round nuclei that included small nucleoli (Figure 2). We also observed eosinophil-rich infiltrates in the tumor nests, tubular structures containing periodic acid-Schiff-positive mucus in the lumen, goblet cells, and venous invasion (Figure 3). The specimen was lined by normal muscular tissue.

We performed immunostaining of the resected specimen. The ductal structure was positive for epithelial membrane antigen (EMA) and carcinoembryonic antigen (CEA), and the solid structure was positive for cytokeratin. The mindbomb E3 ubiquitin protein ligase 1 (MIB-1)-positivity rate was 7.9%. There was no histological capsule; the macroscopic capsule-like structure observed may have been connective tissue covering the margin of the resected specimen.

On histopathology, the tumor was diagnosed as SMECE originating from the minor salivary glands of the lip. Based on its MIB-1-positivity rate, we designated the malignancy of the tumor as intermediate. On microscopy, the tumor nests were close to the surgical margin: 1mm at the nearest point. However, additional surgery was not performed at our patient’s request. We performed contrast-enhanced CT of his neck and scintigraphy of his whole body, neither of which revealed any metastases. Considering the potential for metastasis from the thyroid gland or autoimmune disease, blood tests were also performed. He had no abnormalities in his thyroid hormone levels (tri-iodothyronine, thyroxin and thyroid-stimulating hormone), autoantibody levels (antinuclear antibody, anti-Sjögren’s syndrome antigens A and B), or whole-blood eosinophil count. Nevertheless, we closely monitored our patient, with regular check-ups every three months and contrast-enhanced CT imaging of his cervical region once every year.

**Figure 4:**
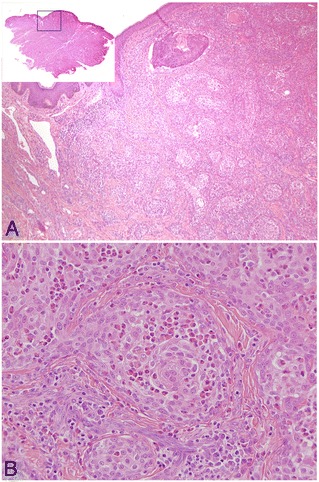
**Histological features of the recurrent tumor. (A)** The density of the fibrous connective tissue was lower than that of the first specimen (hematoxylin and eosin (H&E) staining; macroscopic view, ×100). **(B)** Tumor-associated tissue eosinophilia and fibrous stroma were also evident in the recurrent tumor (H&E; ×200).

Three years after the first resection, a mass measuring 2mm in diameter was detected in the same region. Local recurrence was suspected, and the tumor was resected with a 5mm safety margin. Again, the resected specimen was diagnosed on histopathology as SMECE. Its histologic features were similar to those of the primary tumor (Figure 4), with a lower density of fibrous connective tissue. This specimen also had tumor-free margins on histology. Its MIB-1 positivity rate was 5.8%.

A physical examination and contrast-enhanced CT of his neck revealed no signs of recurrence or metastasis three and a half years after the second surgery.

## Discussion

SMECE is a rare variant of thyroid carcinoma, and only approximately 30 cases have been reported to date. The tumor is a low-grade malignancy with a good prognosis [2]. In 1987, Chan and Saw [3] reported a low-grade MEC showing an unusual pattern of extensive central sclerosis in the parotid gland and termed it sclerosing MEC. In their case, tissue eosinophils were not present. Since then, 20 cases of tumors in the major salivary glands and three of tumors in the minor salivary glands have been reported [3-15]. In many of these cases, marked eosinophilic infiltration was observed (Table 1). In the latest World Health Organization classification [16], the sclerosing variant of MEC was mentioned; however, eosinophilia associated with MEC was not addressed.

**Table 1:** **Past reports of sclerosing mucoepidermoid carcinoma**

**Source**	**Age (years)**	**Gender**	**Site**	**Size (cm)**	**Grade**	**Treatment**	**Follow-up**	**TATE**
*Chan* and *Saw* [3]	36	F	Parotid gland	6×3×2	Low	Superficial parotidectomy	NA	−
*Muller et al*. [4]	17	F	Parotid gland	2	Intermediate	Total parotidectomy	NA	+
*Muller et al.* [4]	60	F	Parotid gland	4.5×4	Intermediate	Resection + RT	NA	+
*Sinha et al.* [5]	65	M	Minor salivary glands (parapharyngeal space)	5×4×3	High	Resection +RT	NA	−
*Urano et al*. [6]	57	F	Parotid gland	2.5×2	Low	Superficial parotidectomy	Lymph node metastasis/3y	+
*Urano et al*. [6]	43	M	Submandibular gland	4.5×2.5	Low	Total excision of submandibular gland	Dead of disease/7y	+
*Fadare et al*. [7]	44	F	Parotid gland	4×2	Low	Total parotidectomy + RT	NED/7y	−
*Ide et al*. [8]	28	M	Minor salivary glands (retromolar pad)	2×2	Intermediate	Total excision	NA	+
*Heavner et al*. [9]	23	F	Parotid gland	2×1	Low	Total parotidectomy + RT	NED/1y	+
*Veras et al*. [10]	70	F	Parotid gland	4×3	Low	Superficial parotidectomy	Dead of other disease/11y	+
*Veras et al.* [10]	37	M	Parotid gland	2.2×1×1	Low	Superficial parotidectomy	NED/17y	+
*Veras et al*. [10]	49	F	Parotid gland	2.6×1.7	Low	Superficial parotidectomy	NED/4mo	+
*Veras et al*. [10]	16	F	Parotid gland	2×2	Intermediate	Superficial parotidectomy	NED/10mo	+
*Aguiar et al*. [11]	43	F	Minor salivary glands (palate)	4×4	Low	Partial maxillectomy	NED/19mo	+
*Shinhar* [12]	57	F	Parotid gland	2	Intermediate	Superficial parotidectomy + RT	NED/3y	−
*Mendelson et al*. [13]	21	F	Parotid gland	2	Low	Total parotidectomy + level-II neck dissection	NED/3y	−
*Tian et al*. [14]	42	F	Parotid gland	1.5	Low	Local excision	NED/19mo	−
*Tian et al*. [14]	52	F	Parotid gland	1.4	Low	Local excision	NED/31mo	−
*Tian et al*. [14]	62	F	Parotid gland	2	Low	Local excision	Lost to follow-up	−
*Tian et al.* [14]	28	M	Parotid gland	1.2	Low	Local excision	NED/8mo	−
*Tian et al*. [14]	65	F	Parotid gland	1.5	Low	Local excision	NED/4mo	−
*Tian et al*. [14]	32	M	Submandibular gland	1.1	Low	Local excision	Lost to follow-up	−
*Tasaki et al*. [15]	79	M	Submandibular gland	5.6	Low	Total excision of submandibular gland	NED/15mo	+
Current case	61	M	Minor salivary glands (upper lip)	1.2×0.9	Intermediate	Resection	Local recurrence/30mo	+

F, female; M, male; mo, month(s); NA, not available; NED, no evidence of disease; RT, radiotherapy; TATE, tumor-associated tissue eosinophilia; y, year(s).

On histopathology, SMECE consists of islands of cancer cells of low nuclear grade, with a sclerotic stroma heavily infiltrated by chronic inflammatory cells and eosinophils. A mixture of mucinous epithelial cells and glandular structures is also evident, and these components can merge with the squamoid islands or form discrete tubules [17].

Differential diagnoses of our case would include squamous cell carcinoma, adenosquamous cell carcinoma, low-grade cribriform cystadenocarcinoma and microcystic adnexal carcinoma. Immunostaining for markers such as EMA and CEA was useful for diagnosis. We designated the malignancy of the first resected tumor as intermediate on the basis of venous invasion and the MIB-1-positivity rate.

From an epidemiological perspective, previous reports of SMECE in the salivary glands have described its features as similar to those of ordinary MEC, with more frequent occurrence in women (16 of 24 reported cases, including ours), a wide age distribution (16 to 79 years) and predominant occurrence in the parotid glands (17 cases).

To the best of our knowledge, this is the first case of SMECE in the minor salivary glands of the lip; it is also the first case of local recurrence. Most reported cases had a good therapeutic outcome, although one patient developed lymph node metastasis and one died from pulmonary metastasis [6]. According to our survey of past reports, tumor-associated tissue eosinophilia is not necessarily related to malignancy. In addition, cases in which secondary metastasis occurred had a low grade of malignancy. Although local excision was effective in most cases, our experience suggests that SMECE has invasive characteristics.

Many previous reports have classified tumors with eosinophilic infiltration into carcinomas and lymphomas. In past studies, tissue eosinophils have been suggested to indicate an inflammatory host response against a tumor or to be an eosinophilotactic factor derived from tumor cells themselves; however, the mechanism underlying eosinophilic infiltration remains unclear [18,19]. In addition, reports on the prognosis of tumors with eosinophilic infiltration are inconsistent [20,21]. In some hematopoietic tumors, such as Hodgkin’s disease or mucosa-associated lymphoid tissue, lymphoma tissue fibrosis has also been observed [22].

Samoszuk [23] showed that eosinophils stimulate DNA synthesis in fibroblasts, and may be involved in the remodeling of host connective tissue and blood vessels in response to a growing tumor. The tissue sclerosis and venous invasion evident in our case were probably a result of eosinophilic infiltration.

Whether SMECE in the salivary glands represents a distinct tumor type or merely a morphological variant of conventional MEC remains controversial [17]. In most cases with thyroid involvement, SMECE occurs in the setting of Hashimoto’s thyroiditis. However, consistent with findings from other patients with salivary gland involvement, our patient developed no complications such as Sjögren’s syndrome or other autoimmune diseases.

In this case, we resected the tumor with no consideration of the possibility of malignancy and subsequently encountered recurrence three years later. Although it is probable that the secondary tumor originated from residual tumor cells, its growth was slow. It was necessary to perform the second surgery as soon as the diagnosis was made because the surgical margin was close to the tumor.

## Conclusion

We reported a case of local recurrence of SMECE. Our experience with the present case indicates that SMECE can originate in the minor salivary glands and that this rare variant of thyroid carcinoma may be clinically or pathologically misdiagnosed as other conditions, such as inflammatory lesions, benign metaplasia, or different types of malignant tumor with tissue sclerosis. Although the prognosis of SMECE is relatively good according to published reports, we continue to monitor our patient carefully.

## Consent

Written informed consent was obtained from the patient for publication of this case report and accompanying images. A copy of the written consent is available for review by the Editor-in-Chief of this journal.

## Abbreviations

CEA: carcinoembryonic antigen
CT: computed tomography
EMA: epithelial membrane antigen
MEC: mucoepidermoid carcinoma
MIB-1: mindbomb E3 ubiquitin protein ligase 1
SMECE: sclerosing mucoepidermoid carcinoma with eosinophilia
